# The impact of epicardial adipose tissue in patients with acute myocardial infarction

**DOI:** 10.1007/s00392-021-01865-4

**Published:** 2021-05-12

**Authors:** Christoph Fisser, Stefan Colling, Kurt Debl, Andrea Hetzenecker, Ulrich Sterz, Okka W. Hamer, Claudia Fellner, Lars S. Maier, Stefan Buchner, Michael Arzt

**Affiliations:** 1 Department of Internal Medicine II, University Hospital Regensburg, Regensburg, Germany; 2 Department of Pneumology, Donaustauf Hospital, Donaustauf, Germany; 3 Department of Internal Medicine III, University Hospital Regensburg, Regensburg, Germany; 4 Institute for Radiology, University Hospital Regensburg, Regensburg, Germany; 5 Department of Internal Medicine II, Sana Clinics Cham, Cham, Germany

**Keywords:** Epicardial adipose tissue, Acute myocardial infarction, Microvascular obstruction, ST-deviation, Infarct size, Myocardial salvage index

## Abstract

**Aims:**

Epicardial adipose tissue (EAT) has been linked to impaired reperfusion success after percutaneous coronary intervention (PCI). Whether EAT predicts myocardial damage in the early phase after acute myocardial infarction (MI) is unclear. Therefore, we investigated whether EAT in patients with acute MI is associated with more microvascular obstruction (MVO), greater ST-deviation, larger infarct size and reduced myocardial salvage index (MSI).

**Methods and results:**

This retrospective analysis of a prospective observational study including patients with acute MI (*n* = 54) undergoing PCI and 12 healthy matched controls. EAT, infarct size and MSI were analyzed with cardiac magnetic resonance imaging, conducted 3–5 days and 12 weeks after MI. Patients with acute MI showed higher EAT volume than healthy controls (46 [25.;75. percentile: 37;59] vs. 24 [15;29] ml, *p* < 0.001). The high EAT group (above median) showed significantly more MVO (2.22 [0.00;5.38] vs. 0.0 [0.00;2.18] %, *p* = 0.004), greater ST-deviation (0.38 [0.22;0.55] vs. 0.15 [0.03;0.20] mV×10^−1^, *p* = 0.008), larger infarct size at 12 weeks (23 [17;29] vs. 10 [4;16] %, *p* < 0.001) and lower MSI (40 [37;54] vs. 66 [49;88] %, *p* < 0.001) after PCI than the low EAT group. After accounting for demographic characteristics, body-mass index, heart volume, infarct location, TIMI-flow grade as well as apnea–hypopnea index, EAT was associated with infarct size at 12 weeks (B = 0.38 [0.11;0.64], *p* = 0.006), but not with MSI.

**Conclusions:**

Patients with acute MI showed higher volume of EAT than healthy individuals. High EAT was linked to more MVO and greater ST-deviation. EAT was associated with infarct size, but not with MSI.

**Graphic abstract:**

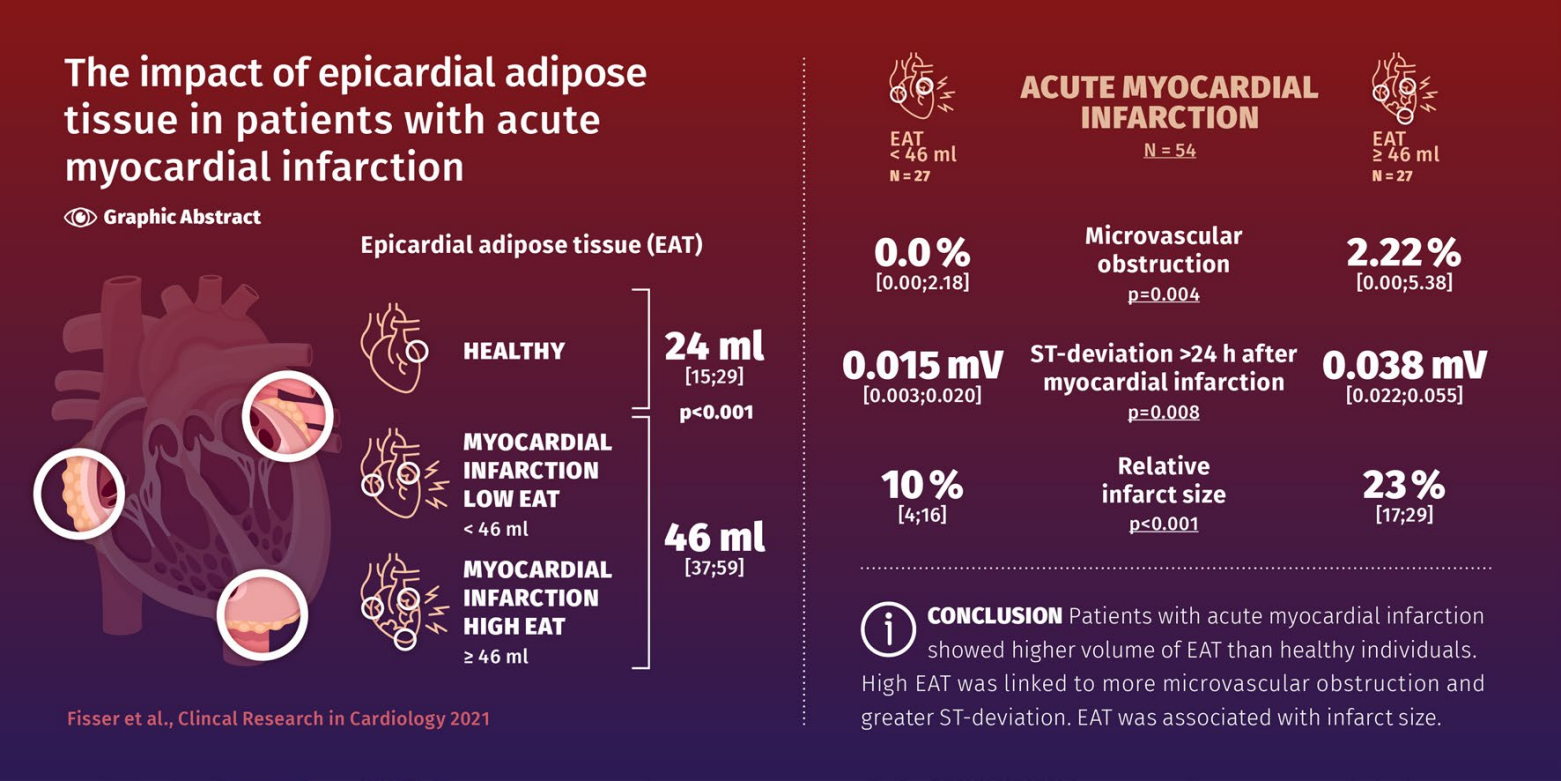

## Introduction

Besides traditional cardiovascular risk factors, body fat is an important factor for determining cardiometabolic risk [1]. The distribution rather than the absolute quantity of excess body fat plays a key role in the pathophysiology of cardiovascular disease [2]. Growing evidence suggests that epicardial adipose tissue (EAT) has been linked to impaired reperfusion success after percutaneous coronary intervention (PCI) [3]. Increased EAT is associated with incident myocardial infarction (MI) [4] and with angiographic severity of an acute coronary syndrome [5]. From previous studies it is known that infarct size [6, 7] is an important predictor of major cardiovascular endpoints in patients after ST-elevation myocardial infarction (STEMI) [7–9]. Cardiac magnetic resonance imaging (CMR) is the gold standard for assessing infarct size [10] and EAT can be assessed as well. Furthermore, EAT-induced elevation of paracrine mediator levels may activate platelet aggregation, thereby increase microvascular obstruction (MVO) [11, 12] and thus may result in larger infarct size and myocardial salvage index (MSI). On the other hand, Bière [13] and Gohbara [14], reported a protective effect of EAT on infarct size and MSI.

Therefore, the aim of this observational study was to investigate whether EAT in patients with acute MI is associated with more MVO, greater ST-deviation, larger infarct size and reduced MSI.

## Methods

### Patients and participants

**Fig. 1:**
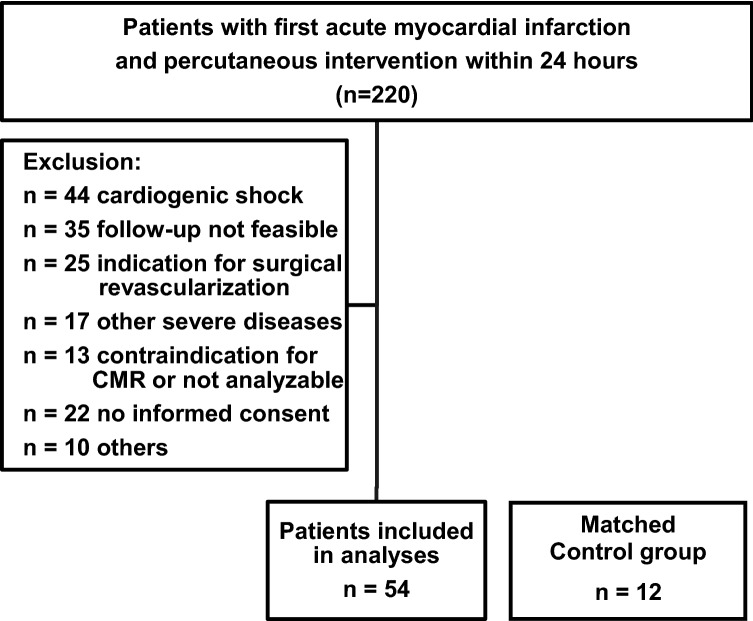
Flowchart of the retrospective analysis of a prospective observational study

This is a retrospective analysis of a prospective observational study published previously [15]. Two hundred twenty consecutive patients aged between 18 and 80 years with a first acute MI who were treated by PCI within 24 h after symptom onset at the University Medical Center Regensburg were tested for eligibility (Fig. 1) [15]. Acute MI was defined as new ST elevation on the electrocardiogram or as native coronary artery occlusion. Key exclusion criteria were previous MI or previous myocardial revascularization, indication for surgical myocardial revascularization, cardiogenic shock, and contraindications for CMR (e.g. previous cardiac device implantation), including in the final sub-analysis 54 patients with acute MI (Fig. 1). As a control group, 12 healthy matched (age, sex, BMI) volunteers without any history of heart disease (potential living kidney donors) [16] underwent CMR (Fig. 1).

The local Ethics Committee approved the study protocol based on the regulations stated in the Helsinki Declaration of Good Clinical Practice. Prior to enrolment, written informed consent was obtained from each participant.

### Percutaneous coronary intervention

Each patient received medication according to current guidelines. PCI was conducted according to standard clinical practice [15]. In addition, administration of glycoprotein IIb/IIIa inhibitors and thrombectomy were at the discretion of the interventionalist.

### Assessment of outcomes

Patients with acute MI underwent CMR and polysomnography 3–5 days and 12 weeks after PCI [15, 17, 18]. In a subset of STEMI patients with analyzable [19] and available routine electrocardiograms (ECG) before PCI and > 24 h after PCI (*n* = 27), a non-prespecified sub-analysis of ST-deviations was performed as described previously [20].

### Cardiac magnetic resonance image analysis

The CMR study protocol has been published previously [15]. Additional details of the image analysis and the acquisition protocol are described in the online supplement.

**Fig. 2:**
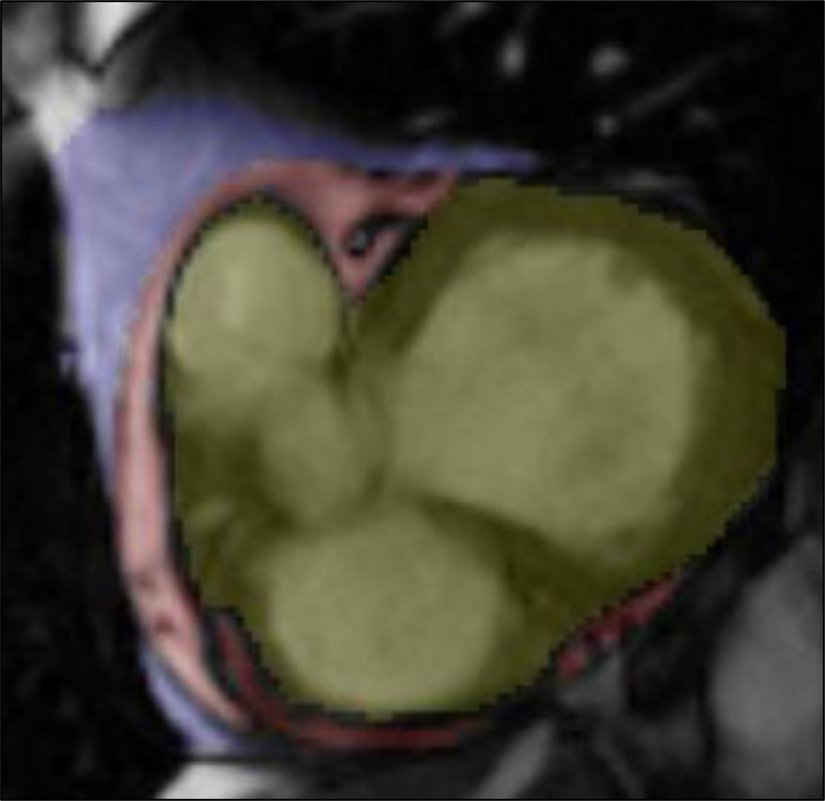
MRI, short-axis slice. Pericardial adipose tissue (blue), epicardial adipose tissue (red), heart volume (yellow)

Cardiac adipose tissue was analyzed by means of the highly accurate and reproducible methods of CMR and the 3-dimensional slice summation technique [21] using short-axis slices in consecutive end-diastolic images [21]. A specific protocol was applied starting with the image on which the mitral valve annulus was first visible down to the apex [21]. Cardiac adipose tissue was divided into EAT and paracardial adipose tissue (Fig. 2). EAT was defined as adipose tissue in-between the pericardium, whereas, paracardial adipose tissue was specified as adipose tissue outside the pericardium but directly adherent to the heart. The sum of epicardial and paracardial adipose tissue was referred to as pericardial adipose tissue [22]. EAT and paracardial adipose tissue were manually segmented with the free software ITK Snap (published under General Public License) [23]. Heart volume without cardiac adipose tissue was quantified by manually tracing the area in-between the visceral layer of the pericardium. MI was assumed, if the signal intensity of hyperenhanced myocardium on delayed enhancement imaging was > 5 standard deviations above the mean signal intensity of the remote region and MVO was defined as a hypoenhanced region within the infarcted myocardium [24, 25]. Infarct size and MVO were expressed as a percentage of the total left ventricular myocardial volume [20], when not stated otherwise. The MSI represents the difference between the area at risk and the final MI size highlighting the amount of myocardium which can be saved [15].

### Statistical analysis

Categorical data are presented as frequency with percentages, and comparisons between categorical variables were made with the Chi-squared test. Normally distributed quantitative data are expressed as mean ± standard deviation, non-normal as median and interquartile range (IQR). Comparisons between quantitative variables were done with either unpaired Student’s t test or Mann–Whitney *U* test as necessary. Consecutive measurements were analyzed with the paired *t*-test.

MVO was classified as present, if > 0% of left ventricular volume were affected. Associations between EAT, ST-deviation and infarct size or MSI were described using linear regression analysis. EAT was included as continuous variable. Multivariate linear regression analyses were conducted to identify predictors of infarct size at baseline and after 12 weeks as well as MSI, including known possible predictors such as infarct location, TIMI flow grade before and after percutaneous coronary intervention and sleep-disordered breathing as previously published [15, 26]. All models were corrected for baseline characteristics such as age, sex, body-mass index and heart volume. A two-sided *p*-value of < 0.05 was considered statistically significant. Data entry and calculations were made with the software package SPSS 25.0 (Chicago, EUA) and R (version 2.14.2).

## Results

### Study population

54 patients fulfilled the inclusion criteria and were enrolled in the study (Fig. 1). The baseline characteristics such as age, sex, body weight, blood pressure were similarly distributed between patients with acute MI (MI group) and the control group, except for heart rate that was lower in the latter group (Table 1).

**Table 1:** Demographics and baseline characteristics: healthy controls and patients with acute myocardial infarction

	Control group (*n* = 12)	MI group (*n* = 54)	*p*-value
Age, years	52 ± 10	55 ± 10	0.406
Men, *n* (%)	9 (75)	46 (85)	0.392
BMI, kg/m^2^	26.6 ± 2.4	28.4 ± 3.6	0.107
Body surface area, m^2^	1.9 ± 0.2	2.0 ± 0.2	0.079
Systolic blood pressure, mmHg	131 ± 14	131 ± 21	0.903
Diastolic blood pressure, mmHg	83 ± 8	80 ± 12	0.362
Heart rate, min^−1^	63 ± 7	75 ± 17	**0.001**

Data are expressed as *n* (%) or mean ± standard deviation, *BMI* body mass index, Control group matched for age, sex and BMI, significant *p* values (*p* < 0.05) marked in bold

### Cardiac adipose tissue

**Fig. 3:**
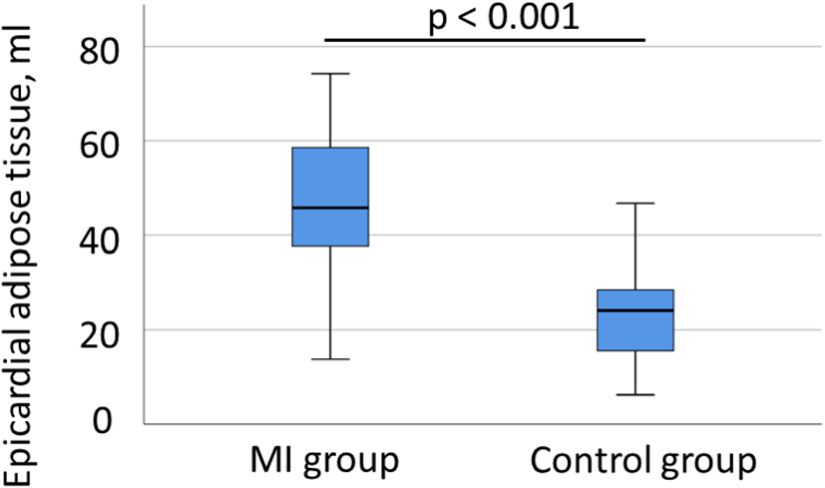
Box plot of epicardial adipose tissue in patients with myocardial infarction (MI group) versus healthy control group. Data are expressed as median, minimum, maximum, 25. percentile, and 75. percentile

Patients with acute MI had a significantly higher EAT volume (46 [25./75. percentile: 37; 59] vs. 24 [15; 29] ml, *p* < 0.001; Fig. 3) and total cardiac adipose tissue (163 [132; 201] vs. 117 [84; 159] ml, *p* < 0.001) than the control group. The amount of paracardial adipose tissue did not significantly differ between the MI and the control group (113 [90; 146] vs. 99 [69; 124] ml, *p* = 0.126). Same results were seen for EAT, total cardiac adipose tissue and paracardial adipose tissue divided by body-mass index (1.6 [1.3; 2.1] vs. 0.9 [0.6; 1.1] ml/kg/m^2^, *p* < 0.001; 5.7 [5.0; 6.7] vs. 4.5 [3.4; 5.9] ml/kg/m^2^, *p* = 0.029; 4.0 [3.3; 4.7] vs. 3.7 [2.7; 4.7] ml/kg/m^2^, *p* = 0.435, respectively).

### Patients with acute myocardial infarction

The baseline characteristics of the two groups of patients stratified according to the median EAT volume at baseline are depicted in Table 2. The two groups did not differ with respect to age, height, heart rate, systolic or diastolic blood pressure and total sleep time. The high EAT group showed significantly higher body mass index, AHI, heart volume, maximum creatine-kinase and proportion of men than the low EAT group. The two groups did not differ with regard to cardiovascular risk factors, such as cholesterol, low density lipoprotein (LDL), high density lipoprotein (HDL), triglycerides, CRP levels, smoking status, or the prevalence of diabetes mellitus. Both groups were similar with respect to infarct type, infarct location, or in TIMI flow rate before and after PCI.

**Table 2:** Demographics and baseline characteristics according to epicardial adipose tissue of patients with acute myocardial infarction

	EAT < 46 ml (*n* = 27)	EAT ≥ 46 ml (*n* = 27)	*p*-value
Age, years	53 ± 11	57 ± 9	0.139
Men, *n* (%)	20 (74)	26 (96)	**0.022**
BMI, kg/m^2^	27 ± 3	30 ± 3	**0.010**
Systolic blood pressure, mmHg	131 ± 21	131 ± 23	0.985
Diastolic blood pressure, mmHg	80 ± 11	79 ± 13	0.867
Heart rate, min^−1^	73 ± 15	76 ± 20	0.533
AHI, h^−1^	11 ± 13	28 ± 21	**0.001**
Diabetes mellitus, *n* (%)	4 (15)	5 (19)	0.715
Positive smoking status, *n* (%)	22 (82)	17 (63)	0.129
Maximum creatine-kinase, U/l^a^	1089 [712; 1794]	2687 [1147; 4021]	**0.004**
LDL, mg/dl	127 ± 40	119 ± 25	0.404
HDL, mg/dl	43 ± 15	43 ± 12	0.857
Cholesterol, mg/dl	193 ± 46	185 ± 27	0.480
Triglycerides, mg/dl	164 ± 92	159 ± 88	0.838
CRP, mg/l^a^	5 [2; 11]	7 [3; 25]	0.256
Symptom-to-balloon time, h	8 ± 8	7 ± 7	0.420
NSTEMI, *n* (%)	7 (26)	3 (11)	0.161
TIMI flow grade before PCI, *n* (%)			0.444
Grade 0	22 (81)	24 (89)	
Grade 1	5 (19)	3 (11)	
TIMI flow grade after PCI, *n* (%)			0.299
Grade 2	1 (4)	3 (11)	
Grade 3	26 (96)	24 (89)	
Heart volume, ml^a^	483 [425; 558]	532 [468; 683]	**0.014**
Epicardial adipose tissue, ml^a^	37 [29; 41]	59 [52; 69]	** < 0.001**

Data are expressed as *n* (%) or mean ± standard deviation unless otherwise stated, *BMI* body mass index, *AHI* apnea–hypopnea-index, *LDL* low density lipoprotein, *HDL* high density lipoprotein, *NSTEMI* non-ST segment elevation myocardial infarction, *TIMI* Thrombolysis in Myocardial Infarction, *CRP* C reactive protein

^a^Data are expressed as median (25. percentile; 75. percentile), significant *p* values (*p* < 0.05) marked in bold

### Microvascular obstruction and ST-deviation

**Fig. 4:**
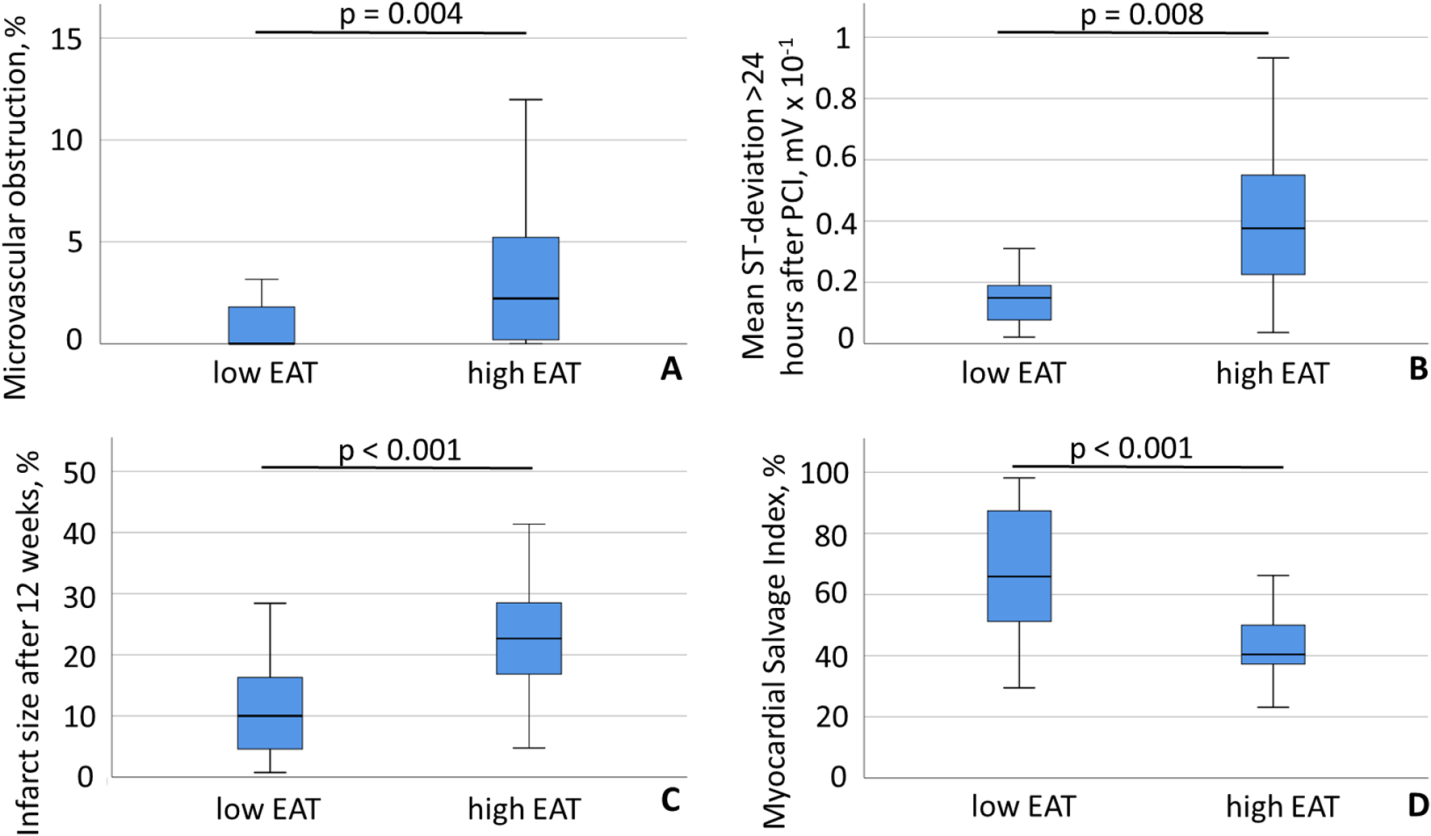
Box plots of microvascular obstruction **a**, mean ST-deviation > 24 h after percutaneous coronary intervention (PCI) **b**, infarct size after 12 weeks **c** and myocardial salvage index **d** according to the low and high epicardial adipose tissue (EAT) volume group. Data are expressed as median, minimum, maximum, 25. percentile, and 75. percentile

12 weeks after PCI, the high EAT group showed significantly more MVO than the low EAT group (2.22 [0.00; 5.38] vs. 0.0 [0.00; 2.18] %, *p* = 0.004; Fig. 4a). The best predictive EAT volume for MVO was 40.8 ml (sensitivity 77.4%, specificity 60.9%). MVO > 0% was present in 31 of 54 patients (57%) and the extent of MVO was significantly associated with the extent of EAT (B = 0.324 [0.024; 0.234], *p* = 0.017) in univariate analysis, but not after accounting for potential confounders.

In a subset of STEMI patients with analyzable routine ECG (*n* = 27), the high and the low EAT groups had similar ST-deviations before PCI (1.13 [0.73; 1.87] vs. 1.00 [0.39; 1.19] mVx10^−1^, *p* = 0.294). However, > 24 h after PCI, patients from the high EAT group had significantly greater ST-deviations compared to the low EAT group (0.38 [0.22; 0.55] vs. 0.15 [0.03; 0.20] mVx10^−1^, *p* = 0.008; Fig. 4b). ST-deviation > 24 h after PCI was significantly associated with infarct size 12 weeks after PCI (B = 29.26 [17.86; 40.66] *p* < 0.001, Fig. S1).

### Predictors of infarct size and myocardial salvage index

The relative infarct size in relation to the left ventricular volume at baseline and after 12 weeks was significantly higher in the high EAT group than in the low EAT group (24 [16; 34] vs. 15 [7; 22] %, *p* = 0.003; 23 [17; 29] vs. 10 [4; 16] %, *p* < 0.001; Fig. 4c, respectively). EAT and infarct size were significantly correlated at baseline and at 12 weeks (*R*^2^ linear = 0.220, *p* < 0.001, *R*^2^ linear = 0.302, *p* < 0.001; Fig. S2a, b). EAT was an independent predictor of infarct size at baseline and at 12 weeks size after accounting for potential confounders such as infarct location, TIMI flow grade before and after PCI and sleep-disordered breathing, respectively (B = 0.49 [0.0; 0.90], *p* = 0.017; B = 0.38 [0.11; 0.64] *p* = 0.006; Table 3, respectively). When including only those with high EAT and low PAT (*n* = 8) and those with low EAT combined with high PAT (*n* = 7), univariate analysis revealed no association with baseline and 12 weeks follow-up infarct size.

**Table 3:** Multivariate linear regression analysis: EAT and infarct size at baseline, 12 weeks and myocardial salvage index

Predictors of endpoint	Infarct size (baseline), g B (95% CI)	*p*-value	Infarct size, g (12 weeks) B (95% CI)	*p*-value	Myocardial salvage index, g B (95% CI)	*p*-value
Anterior infarction	7.17 ( – 5.18; 19.52)	0.248	1.49 ( – 6.70; 9.68)	0.715	0.02 ( – 0.08, 0.12)	0.645
TIMI-flow pre PCI*	11.71 ( – 3.85; 27.26)	0.136	6.69 ( – 3.63; 17.01)	0.198	– 0.02 ( – 0.14, 0.11)	0.761
TIMI-flow post PCI**	– 2.24 ( – 23.63; 19.15)	0.834	3.25 ( – 10.94; 17.43)	0.646	– 0.11 ( – 0.28, – 0.06)	0.214
AHI***	3.07 ( – 9.13; 15.26)	0.614	9.39 (1.20; 17.38)	**0.025**	– 0.20 ( – 0.30, – 0.10)	** 0.001**
Epicardial adipose tissue, ml	0.49 (0.09; 0.90)	**0.017**	0.38 (0.11; 0.64)	**0.006**	0.00 (0.00, 0.00)	0.420
Time to balloon, min	0.11 ( – 0.61; 0.84)	0.751	0.17 ( – 0.31; 0.65)	0.477	0.00 ( – 0.01; 0.01)	0.771
Diabetes mellitus****	4.78 ( – 9.60; 19.16)	0.506	4.86 ( – 4.68; 14.40)	0.310	– 0.06 ( – 0.17; 0.06)	0.313
Model summary*****	*R*^2^ = 0.478, F = 3.503		*R*^2^ = 0.604, F = 5.828		*R*^2^ = 0.524, F = 4.195	

*AHI* apnea–hypopnea-index, *EAT* epicardial adipose tissue, *B* regression coefficient, *CI* confidence interval, *TIMI* Thrombolysis in Myocardial Infarction

*TIMI grade 0 vs TIMI grade 1

**TIMI grade 2 vs TIMI grade 3, *PCI* percutaneous coronary intervention

***AHI, stratified in ≥ 15/h and < 15/h

****Diabetes mellitus yes and no

*****All models controlled for age, sex, BMI and heart volume, significant *p* values (*p* < 0.05) marked in bold

The MSI was significantly lower in the high EAT group compared to the low EAT group (40 [37; 54] vs. 66 [49; 88] %, *p* < 0.001; Fig. 4d). In univariate linear regression analysis EAT was significantly associated with MSI (B =  – 0.005 [ – 0.007;  – 0.002], *p* = 0.002), however, this association was no longer significant either after adjustment for AHI only (B =  – 0.001 [ – 0.004; 0.001], *p* = 0.262) or in the fully adjusted model (Table 3).

## Discussion

The current retrospective analysis of a prospective observational study on the association between EAT and MI size yielded several novel findings. First, patients with acute MI had significantly more EAT than healthy individuals.

Second, patients with acute MI and high EAT had more MVO than patients with low EAT. Before PCI, ST-deviation was similar between groups. However, after PCI, the high EAT group had more residual ST-deviation compared to the low EAT group. ST-deviation after PCI was associated with infarct size.

Third, patients with acute MI and high EAT had larger infarct size at baseline and 12 weeks after MI than those with low EAT. EAT was significantly associated with infarct size at baseline and 12 weeks after MI, independently of demographic characteristics, infarct location, efficacy of PCI as well as sleep-disordered breathing. The high EAT group had a significantly lower MSI.

### EAT in patients with acute myocardial infarction and healthy controls

Patients with acute MI had 50% more EAT in comparison to healthy controls. Similarly, Mahabadi et al. [4] (Table S1) observed in a cohort study of 4093 participants, that patients with incident coronary events had 21% more EAT compared to patients without coronary events. In addition, hypertensive men without MI had less EAT in comparison to those with MI [27]. Conflicting data from Bière et al. [13] (Table S1) showed no differences in epicardial adipose tissue volumes between STEMI patients (primary PCI and rescue PCI within 12 h) and healthy controls with a very broad spectrum of EAT values (0–105 ml), resulting in 16% patients with MI and less than 10 ml EAT. According to previous results [4], the results from the present analysis and pathophysiology, these findings seem to be disputable. It is uncertain whether such conflicting results are a consequence of differences in the studied patient population or differences in methodology of assessment of EAT (e.g. technique of MRI or interval between MI and MRI) [13]. The effect of EAT on cardiovascular events is thought to result from the endocrine, pro-inflammatory activity of EAT [22].

### EAT, MVO and ST-deviation in patients with myocardial infarction

MVO, assessed by CMR, is regarded as a better predictor for mortality than ejection fraction and different clinical scores [25, 28, 29] and the extent of MVO is associated with short-term and long-term mortality [29, 30]. MVO occurred in 57.4% in the MI cohort reflecting the largest pooled data analysis with 1688 patients on MVO and STEMI with 56.9% [29]. Alam et al. [31] reported an association of EAT and impaired myocardial flow reserve as surrogate parameter for microvascular dysfunction in patients with non-obstructive coronary artery disease. Classifying patients with acute MI in a high and low EAT volume group, we could show that within 12 weeks after PCI high EAT volume was associated with more MVO. But this association was not robust in multivariate analysis. Contrary, Gohbara et al. [14] (Table S1) reported very low amounts of MVO (1%) in patients with low (16 ml) and high (48 ml) EAT volumes. However, in the study of Gohbara et al. [14] BMI was 25 kg/m^2^ and 22% patients had TIMI flow > 1 reflecting a healthier population than in the current cohort. Another study [13] with similar subepicardial adipose tissue volumes in STEMI and healthy controls, reported MVO less frequent in STEMI patients with high EAT (> 34 ml) compared to low EAT (< 34 ml). The current analysis showed that ST deviation was similar before PCI and deviation was greater after PCI in the high EAT group than in the low EAT group. Findings are in line with Zencirci et al. [3] (Table S1), who showed that patients with acute MI with resolution of ST segment deviation ≥ 70% had a lower thickness of EAT compared to those with a poor resolution of ST segment deviation (< 70%).

### EAT, infarct size and MSI in patients with acute myocardial infarction

Keeping in mind that infarct size, measured by CMR, is a strong predictor for all-cause mortality after MI [7], our study shows that EAT is an independent predictor of infarct size, both at baseline as well as after 12 weeks. EAT remained an independent predictor of infarct size even after adjustment for traditional risk factors. EAT was associated with greater infarct size assessed with CMR and with higher creatine kinase as a surrogate for greater infarct size. These findings are supported by the association of EAT with MVO and ST-deviation. Both parameters mirroring disturbed microcirculation [32], correlate with infarct size [30, 33].

In contrast to our analyses, others [13, 14] (Table S1) reported conflictive results in STEMI patients with less infarct size in the high EAT group. These findings were stated to be associated with obesity paradox [13, 14]. Bière et al. [13] could not reproduce the established finding that patients with MI have higher EAT volume compared to healthy controls (Table S1) [4, 27].

While EAT was significantly associated with infarct size in this analysis, independently of several other risk factors for infarct size, this was not the case for the association with MSI. Sleep-disordered breathing was one of the strongest confounders for the association between EAT and MSI, confirming previous findings identifying sleep-disordered breathing as a strong predictor for MSI with a high pathophysiologic plausibility to contribute to cardiac damage [15]. Therefore, the pathophysiological effect of sleep-disordered breathing [34] might be stronger than the endocrine effects of EAT. Similar to the results with respect to infarct size, Gohbara et al. [14] reported an opposite association of EAT and MSI than the current analysis.

However, further evidence for the potentially harmful impact of EAT derive from animal studies, showing that resection of EAT decreases progression of cardiovascular disease in coronary arteries [35, 36]. Therefore, future studies in humans with acute MI are necessary to confirm and clarify the potentially protective effect of decreasing EAT volume on cardiovascular disease.

### Pathophysiological considerations

Evidence indicates that the effect of EAT on cardiovascular disease may mainly result from its release of endocrine proinflammatory mediators such as interleukin-6 (IL-6) and TNF-α [37]. EAT produced pro-inflammatory markers could lead to platelet aggregation and increased leucocyte activation followed by intravascular plugging resulting in MVO [38, 39] and impaired coronary flow reserve [40].

A further explanation could be the more pronounced arteriosclerosis and the higher rates of coronary events due to the inherent pro-inflammatory activity of EAT [4, 41, 42]. On the one side high EAT volume has been shown to be associated with the vulnerability of coronary artery plaques [43] and on the other side severe coronary artery disease may stimulate the expression of pro-inflammatory parameters in the EAT [37].

Taken together, these pathomechanisms may result in a vicious circle of high EAT volume, inflammation and arteriosclerosis which may lead to a more pronounced myocardial damage as seen in the current analysis.

## Limitations

This study is limited by its sample size and its design that does not allow to build causal relations. Therefore, further experimental studies and randomized clinical trials are required. The control group was a small group of healthy volunteers [16]. Therefore, a potential selection bias cannot be excluded in total. However, controls and patients were matched for age, sex and BMI. Despite correcting for BMI and AHI [44], we cannot exclude whether the effect of EAT is independent of body fat distribution. Waist-to-hip ratio and visceral obesity were not assessed. Lesion geometry and location of lesion (proximal vs. distal) was not taken into account in the multivariate analysis.

## Conclusion

Patients with acute MI showed higher volume of EAT than healthy individuals. High EAT was linked to more MVO and less ST-deviation. EAT was significantly associated with infarct size, independently of demographic characteristics, BMI, heart volume, infarct location, efficacy of PCI as well as sleep-disordered breathing. EAT was not independently associated with MSI. Further clinical and mechanistic studies are needed to evaluate whether EAT may contribute to myocardial damage in the early phase after myocardial infarction.
