# Correction to: The impact of epicardial adipose tissue in patients with acute myocardial infarction

**DOI:** 10.1007/s00392-021-01889-w

**Published:** 2021-06-14

**Authors:** Christoph Fisser, Stefan Colling, Kurt Debl, Andrea Hetzenecker, Ulrich Sterz, Okka W. Hamer, Claudia Fellner, Lars S. Maier, Stefan Buchner, Michael Arzt

**Affiliations:** 1 Department of Internal Medicine II, University Hospital Regensburg, Regensburg, Germany; 2 Department of Pneumology, Donaustauf Hospital, Donaustauf, Germany; 3 Department of Internal Medicine III, University Hospital Regensburg, Regensburg, Germany; 4 Institute for Radiology, University Hospital Regensburg, Regensburg, Germany; 5 Department of Internal Medicine II, Sana Clinics Cham, Cham, Germany

## Correction to: Clinical Research in Cardiology (2021) 10.1007/s00392-021-01865-4

The original version of this article, published on May 12, 2021, contained a mistake.

The columns of the graphical abstract were incorrect. The correct information is given below.
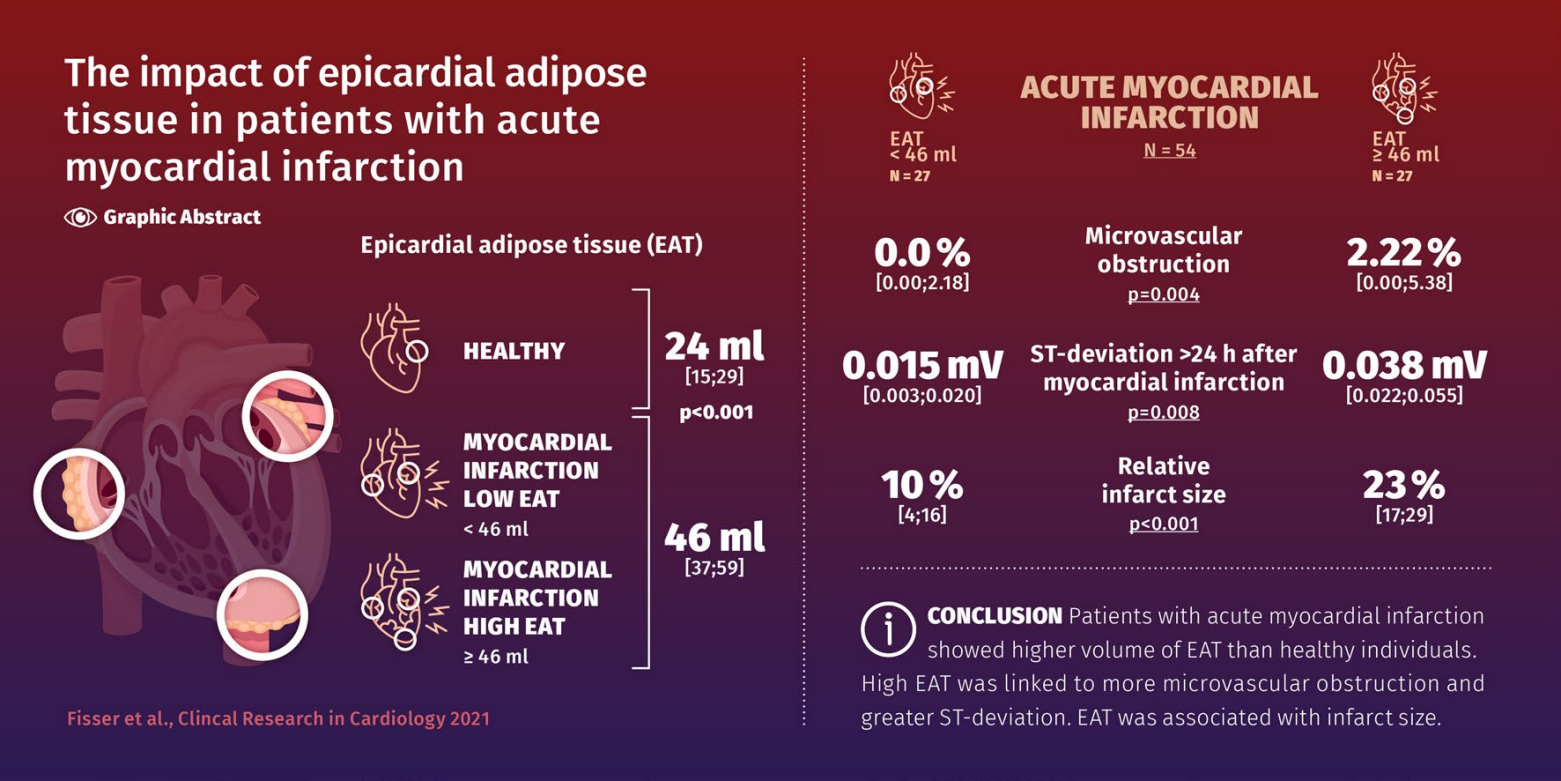


The original article has been corrected.

